# miR-3113-5p, miR-223-3p, miR-133a-3p, and miR-499a-5p are sensitive biomarkers to diagnose sudden cardiac death

**DOI:** 10.1186/s13000-021-01127-x

**Published:** 2021-07-31

**Authors:** Fengping Yan, Yuanyuan Chen, Xing Ye, Fu Zhang, Shiquan Wang, Le Zhang, Xiaoting Luo

**Affiliations:** 1 Department of Forensic Medicine, School of Basic Medical Sciences, Gannan Medical University, 1 Yixueyuan Road, Zhanggong District, Ganzhou, Jiangxi 341000 PR China; 2 Key Laboratory of Prevention and treatment of cardiovascular and cerebrovascular diseases of Ministry of Education, Gannan Medical University, 1 Yixueyuan Road, Zhanggong District, Ganzhou, Jiangxi 341000 People’s Republic of China; 3 Criminal Technology Center of Guangdong Province Public Security Bureau, Guangzhou, Guangdong 510050 PR China; 4 Forensic Science Center of Gannan Medical University, 1 Yixueyuan Road, Zhanggong District, Ganzhou, Jiangxi 341000 People’s Republic of China; 5 Department of Biochemistry and Molecular Biology, School of Basic Medical Sciences, Gannan Medical University, 1 Yixueyuan Road, Zhanggong District, Ganzhou, Jiangxi 341000 PR China

**Keywords:** miRNA, Sudden cardiac death, Molecular pathology, Biomarkers

## Abstract

**Background:**

Sudden cardiac death (SCD) remains a great health threat and diagnostic challenge, especially those cases without positive autopsy findings. Molecular biomarkers have been urgently needed for the diagnosis of SCD displaying negative autopsy results. Due to their nature of stability, microRNAs (miRNAs) have emerged as promising diagnostic biomarkers for cardiovascular diseases.

**Methods:**

This study investigated whether specific cardio-miRNAs (miR-3113-5p, miR-223-3p, miR-499a-5p, and miR-133a-3p) could serve as potential biomarkers for the diagnosis of SCD. Thirty-four SCD cases were selected, 18 categorized as SCD with negative autopsy (SCD-negative autopsy) findings and 16 as SCD with positive autopsy (SCD-positive autopsy) findings such as coronary atherosclerosis and gross myocardial scar. Carbon monoxide (CO) intoxication (*n* = 14) and fatal injury death (*n* = 14) that displayed no pathological changes of myocardium were selected as control group, respectively. Histological analyses were performed to reveal the pathological changes and real-time quantitative polymerase chain reaction (RT-qPCR) was used to determine the expression of those miRNAs.

**Results:**

It showed that heart samples from the SCD-negative autopsy group displayed no remarkable difference with regard to the expression of cleaved-caspase3, CD31, and CD68 and the extent of fibrotic tissue accumulation when compared with control samples. The four cardio-miRNAs were significantly up-regulated in the SCD samples as compared with control. When discriminating SCD from controls, receiver operating characteristic (ROC) curve analysis revealed that the areas under the curve (AUC) of these 4 miRNAs were from 0.7839 to 0.9043 with sensitivity of 64.71–97.06% and specificity of 70–100%. Moreover, when discriminating the specific causes of SCD, the four miRNA expressions increased in the heart from the SCD-negative autopsy group as relative to that from the SCD-positive autopsy group, and a combination of two miRNAs presented higher diagnostic value (AUC = 0.7407–0.8667).

**Conclusion:**

miR-3113-5p, miR-223-3p, miR-499a-5p, and miR-133a-3p may serve as independent diagnostic biomarkers for SCD, and a combination of two of these miRNAs could further discriminate detailed causes of SCD.

## Background

Heart serves as a biological pump that circulates blood throughout our bodies and thus supplying us with oxygen and nutrients. Irregular cardiac activities can restrict blood supply and lead to sudden cardiac death (SCD) [1], a condition that is defined as autopsy identification of a cardiac or vascular anomaly as the probable cause of death for seemingly healthy decedents [2]. SCD is one of the major causes of natural death, causing approximately 1 million of adult deaths in China each year, and remains one of the most challenging tasks in cardiology [3]. Coronary artery diseases closely associate with the high incidence of SCD cases, particularly myocardial infarction (MI) [4]. Acute MI (AMI) is defined as myocardial necrosis in a clinical setting consistent with acute myocardial ischemia [5]. AMI is a specific cause of SCD that often claims deaths in a short period. Unfortunately, deaths from AMI often show negative autopsy findings, and it is not easy to accurately diagnose with the current histopathology techniques. Moreover, data suggested that though implantable cardioverter defibrillators (ICD) have being used to prevent SCD in individuals with existing conditions of cardiomyopathy and inherited arrhythmias [6], their survival benefits are limited as only 20–30% even under appropriate therapy [7]. In addition, albeit left ventricular systolic dysfunction and the severity of heart failure symptoms being predictors of SCD [8], a considerable number of SCD events do not have a pre-existing history of depressed ejection fraction or a clinical history of heart failure [9]. Furthermore, some SCD cases cannot be explained even after systemic autopsy and histological examination. These cases have been postulated to die largely from AMI without grossly observable pathology [10]. Therefore, it is an imperative to find sensitive biomarkers that could be used for detection of SCD, particularly SCD with negative autopsy (SCD-negative autopsy) findings.

Currently, mutations of cardiac-related genes have been implicated in the pathogenesis of SCD, such as MYBPC3 [11], ACE [12] and PKP2 [13] . For example, the ACE DD genotype was associated with a 3.35-fold higher risk of AMI, and a significantly higher risk of SCD (odds ratio (OR) = 6.484, 95% confidence interval (CI): 1.036–40.598, *p* = 0.046) [12] . Inflammation is also reported to play a pivotal role in the pathogenesis of atherosclerosis and cardiovascular disease, with c-reactive protein (CRP) emerging as inflammatory biomarker in AMI [14].

In addition to the above macromolecules, noncoding RNAs have recently received great attention for their potential as diagnostic biomarkers [15]. MicroRNAs (miRNAs) are small noncoding RNAs that regulate gene expression at a post-transcriptional level by repressing messenger RNA (mRNA) translation mainly via binding at the complementary 3′-untranslated region [16]. It has been widely recognized that miRNAs can serve as vital players in many biological processes, especially in the cardiovascular diseases [17]. Studies have shown that aberrant miRNAs expression closely related to AMI [18], hypertension [19], atherosclerosis [20], heart failure [21] and arrhythmias [22]. miR-223 enhanced cardiac fibroblasts proliferation, migration, and differentiation, which led to cardiac fibrosis partially via the involvement of RASA1 [23]. miR-133a plays an important role in atherosclerosis by attenuating lipid accumulation via TR4-CD36 pathway in macrophages [24]. miRNA-499 levels were found to be linearly proportional to myocardial damage, with close relationship with AMI [25]. Our previous work has identified miRNA-3113-5p as a novel cardio-miRNA that is significantly increased after cardiac ischemia-reperfusion (I/R) injury in cardiac and circulating levels and miRNA-3113-5p might serve as a stable marker for diagnosis of cardiac I/R injury [26]. All these miRNAs have been identified as originating from the heart or being heart-resided, making them possible candidates for the diagnostic biomarkers of cardiovascular diseases. However, despite a large number of studies in identifying biomarkers to improve SCD diagnostic efficiency, few biomarkers have progressed to clinical use for SCD.

Given miRNAs’ stability and potential role in various cardiovascular events such as SCD, the aim of the present study is to investigate the value of four cardio-miRNAs (miR-3113-5p, miR-223-3p, miR-133a-3p, and miR-499a-5p) in discriminating SCD from controls. Our data showed that each of these miRNAs had considerable sensitivity when discriminating the SCD from non-cardiac deaths, and a combination of two of these miRNAs could further efficiently discriminate detailed causes of SCD.

## Methods

### Human heart samples

For the purpose of this study, we collected four categories of human heart samples that were autopsied at the Department of Forensic Medicine, School of Basic Medical Sciences, Gannan Medical University over the period 2012–2019. A total of 34 cases of subjects who died from SCD were selected. These cases included 18 of SCD-negative autopsy that presented none notable heart changes at autopsy and 16 of SCD with evidence of heart dysfunction (SCD-positive autopsy). The causes of SCD were made after systemic autopsy. SCD-negative autopsy was defined as sudden deaths without gross heart pathology, with or without histological evidence of early myocardial ischemia (i.e. wavy fiber, hyperesonophilia of cardiomyocytes, and contraction band necrosis). SCD-positive autopsy was defined as sudden deaths in clinic plus severe coronary artery stenosis at autopsy, often with gross or microscopic scarring within myocardium. In addition, 14 cases of fatal injury and 14 cases of carbon monoxide (CO) intoxication were selected as the year-matched control cases. In detail, the heart specimens from fatal injury served as negative control due to none myocardial ischemia in such conditions, and the heart specimens from acute CO intoxication were assigned as positive control due to the global, but not heart-restricted, deficiency of oxygen in such circumstances. Patients who had pre-operative chemo- or radiotherapy history were excluded. Any case with heart implantation was also excluded. All of these cases undergone autopsy examination and were also excluded from influence of other potential substances such as alcohol, illicit drugs, or psychoactive drugs. Detailed demographic and case information regarding the four categories of cases were documented in Table 1. Detailed heart pathology of the four groups of cases were recorded in Table 2. The use of human heart samples for the purpose of research was approved by the Ethical Review Board at the School of Basic Medical Sciences, Gannan Medical University.

**Table 1:** Basic information regarding the human fatal cases

Cause of death	Group	n	Male/female	Age (years)	Postmortem Interval (h)	heart weight (g)	Survival time (h)
CO intoxication	Positive control	14	7/7	30.29 ± 3.11	7.29 ± 1.92	298.9 ± 32.71	1.58 ± 0.57
Fatal injury	Negative control	14	9/5	45.00 ± 4.01^**^	8.07 ± 1.96	312.5 ± 20.18	1.41 ± 0.65
SCD-positive autopsy	SCD	16	14/2	62.69 ± 3.06^***##^	3.75 ± 0.66^#^	455.6 ± 10.49^***###^	1.99 ± 0.93
SCD-negative autopsy	SCD	18	14/4	42.11 ± 3.08^*&&&^	5.50 ± 1.27	377.8 ± 27.32^&^	1.89 ± 0.58

*CO* Carbon monoxide. *SCD* Sudden cardiac death. SCD-positive autopsy, sudden cardiac death with positive autopsy (with evidence of heart dysfunction) findings. SCD-negative autopsy, sudden cardiac death with negative autopsy (without gross heart changes at autopsy) findings

^*^p < 0.05, ^**^p < 0.01, and ^***^p < 0.001 vs. CO intoxication. ^#^*p* < 0.05, ^##^*p* < 0.01, and ^###^*p* < 0.001 vs. Fatal injury. ^&^*p* < 0.05 and ^&&&^*p* < 0.001 vs. SCD-positive autopsy

**Table 2:** Heart pathology in the SCD-negative autopsy, SCD-positive autopsy, and control groups (CO intoxication and fatal injury)

Characteristics	CO intoxication	Fatal injury	SCD-negative autopsy	SCD-positive autopsy
Total cases	*n* = 14	*n* = 14	*n* = 18	*n* = 16
Heart pathology	No evident changes	No evident changes	● 38.9% (7 cases) with both interstitial edema, wavy fibers, and hypereosinophilia of cardiomyocytes; ● 33.3% (6 cases) with only interstitial edema and patchy hypereosinophilic cardiomyocytes; ● 16.7% (3 cases) with interstitial edema and some slightly wavy fibers; ● 11.1% (2 cases) without evident gross or histological changes.	● 56.2% (9 cases) with coronary stenosis (50–75% stenosis), calcification and myocardial fibrosis; ● 43.8% (7 cases) with coronary stenosis (> 75% stenosis), severe myocardial fibrosis and disorder.

### Histological evaluation

All histological evaluation was performed using the human heart samples dissected from the apex of each heart. Due to difficulty in obtaining fresh surgical heart samples, we used the autoptic formalin-fixed paraffin-embedded (FFPE) samples for the purpose of this study. Unlike mRNAs or proteins that are prone to degradation and chemical modification, miRNAs are very small in size (~ 22 nt), less likely to be degraded, and easier to recover from FFPE samples, all of which confer to miRNAs high forensic relevance [27]. The extraction of miRNAs from autoptic FFPE samples was commonly documented in publications [28]. After histological staining, independent pathologists were recruited to review the Hematoxylin & Eosin (H&E) staining sections and PicroSirius Red (PSR) staining sections using a multi-head microscope to result in consensus.

For the H&E staining, human heart tissues were immediately fixed in 4% paraformaldehyde for 24 h, followed by embedding in paraffin. Tissues were then sliced into 5 μm-thick heart slices. Then the slices were dewaxed and dehydrated in a gradient alcohol. Slices were then stained with hematoxylin solution for 10 min at room temperature and incubated in 1% hydrochloric-alcohol solution. Then, slices were washed using tap water, stained with eosin solution for 5 min, and separated by 75, 85, 90, 95 and 100% alcohol solutions for 2 min, respectively. Next, the slices were dried and sealed with neutral gums. After H&E staining, the morphological changes were observed and photographed under an optical microscope (Olympus, Tokyo, Japan).

For the PSR staining, human heart slices were deparaffinized to water and then immersed in 0.1% Sirius red staining solution dissolved in picric acid for 1 h. Then slices were washed in acidified water containing 1.5% hydrogen chloride, dehydrated and mounted. Collagen and non-collagen components were red- and orange-stained, respectively.

### Immunohistochemistry (IHC) staining

Heart slices from the four categories were also subject to IHC analysis. Briefly, the slides were incubated in 3% H_2_O_2_ solution for 10 min and antigen retrieval was performed by steam heating in 10 mM citrate buffer (pH 6.0) for 30 min. After epitope recovery, the slides were then treated with 10% of normal goat serum for 60 min, followed by incubation with active caspase 3 antibody (1:500, Cell Signaling Technology, Catlog #9664, MA, USA), CD31 antibody (1:1000, Cell Signaling Technology, Catlog #3528), and CD68 antibody (1:500, Cell Signaling Technology, Catlog #76437) incubation overnight at 4 °C. The slides were washed and incubated with secondary horseradish peroxide (HRP)-linked secondary antibody (Vector Laboratories, MN, USA) at 1:500 dilutions for 1 h. The samples were treated with the chromogen DAB for antigen detection and the final counterstaining was performed with hematoxylin.

### RNA extraction and reverse transcription

All paraffin was removed from the FFPE heart sections by treating with Deparaffinization Solution. The extraction of miRNAs from FFPE tissues was performed using a commercial miRNeasy FFPE kit (Qiagen, Catlog #217504, Hilden, Germany). Briefly, samples were incubated by heat treatment in an optimized lysis buffer, which contains proteinase K, to release RNAs from the sections. Supernatant was collected and treated with a DNase, followed by buffer RBC and ethanol treatments. The cleaned samples were then applied to an RNeasy MiniElute spin column, where the total RNAs including miRNAs, bound to the membrane and contaminants were efficiently washed away. Total RNAs including miRNAs were then eluted in a minimum of 14 μL of RNase-free water. Reverse transcription of the total RNA was performed using a Mir-X miRNA First-Strand Synthesis Kit (Takara, Tokyo, Japan) for RT-qPCR of miRNA. The synthesized cDNA was stored at − 80 °C for later use.

### Real-time quantitative polymerase chain reaction (RT-qPCR)

QuantiFast Multiplex RT-PCR Kit (Qiagen) was used for RT-qPCR analysis. According to the kit instructions, an aliquot of 25 μL reaction system was mixed and amplified using the Applied Biosystem 7500 fluorescence quantitative PCR instrument (Darmstadt, Germany). Primers used for this study were listed in Table 3. The 2^-ΔCt^ method was used to calculate the relative expression of the target genes where ΔCt_miRNA_ = Ct_miRNA_ − C_thousekeeping._

**Table 3:** Primer sequences used in this study

miRNAs	Primer Sequences 5′-3′
hsa-miR-3113-5p	GTCCTGGCCCTGGTCCGGGTCC
hsa-miR-499a-5p	TTAAGACTTGCAGTGATGTTT
hsa-miR-223-3p	TGTCAGTTTGTCAAATACCCCA
hsa-miR-133a-3p	TTTGGTCCCCTTCAACCAGCTG

### Statistical analysis

All data were expressed as mean ± standard error of the mean (SEM). Statistical analysis was performed using independent Student’s *t* test. The diagnostic powers of miR-3113-5p, miR-223-3p, miR-133a-3p, and miR-499a-5p were evaluated by receiver operating characteristic (ROC) analysis. Areas under the curve (AUCs) were calculated. All statistical analyses were performed using GraphPad Prism 8.0 software (La Giolla, CA, USA). *P* < 0.05 was considered as statistically significant.

## Results

### Cases from the SCD-negative autopsy group did not show notable pathological changes

**Fig. 1:**
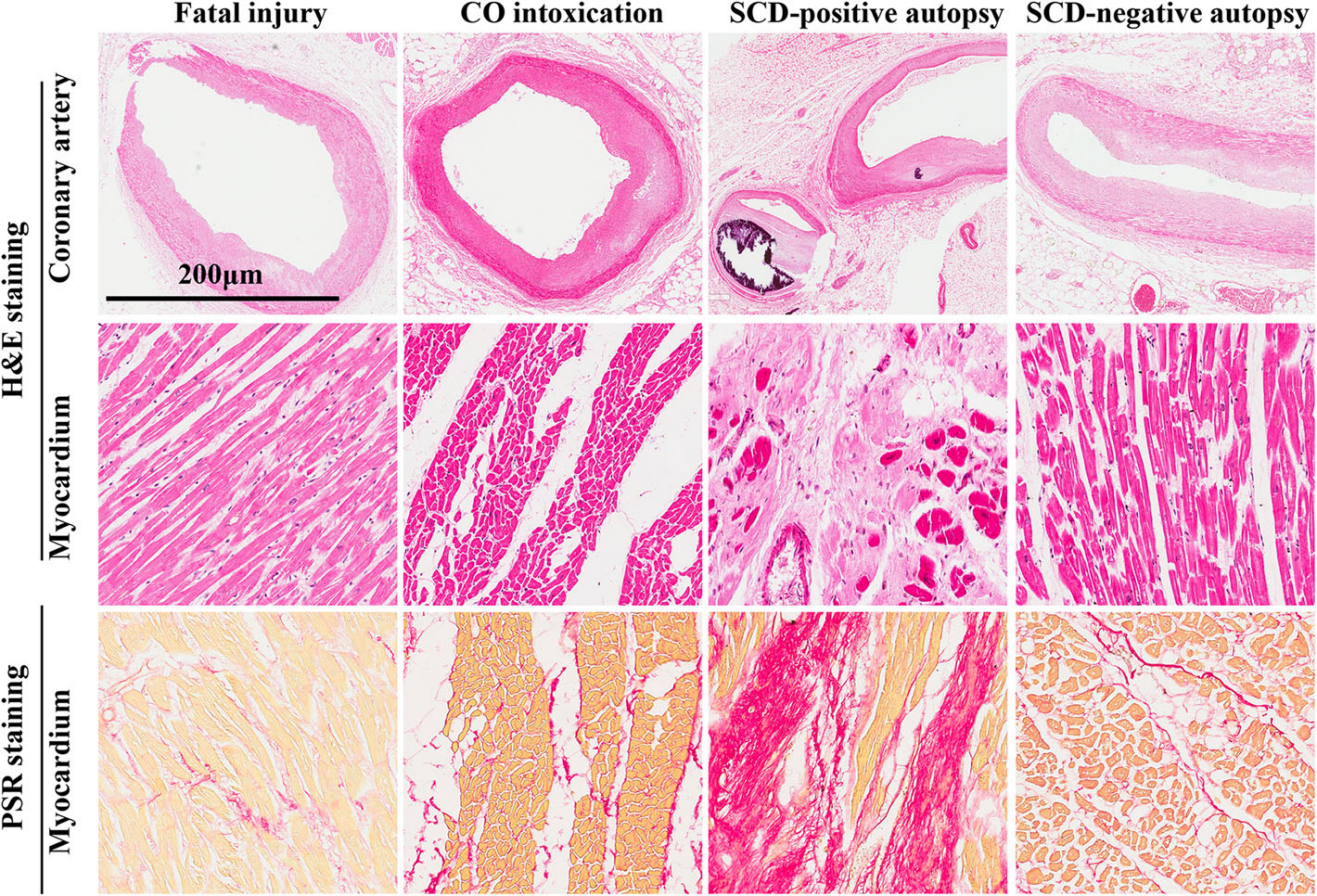
Representative photographs of the morphological changes in the heart tissues of fatal injury, carbon monoxide (CO) intoxication, sudden cardiac death with positive autopsy (SCD-positive autopsy), and sudden cardiac death with negative autopsy (SCD-negative autopsy) by H&E staining and PicroSirius Red (PSR) staining. The SCD-positive autopsy group presented with severe coronary stenosis, calcification, and fibrotic tissue accumulation, whereas the fatal injury and CO intoxication groups did not show notable pathological changes. SCD-negative autopsy group showed slight histological changes such as interstitial edema, fiber rupture, and patchy hypereosinophilia of cardiomyocytes. Scale bar = 200 μm

As shown in Table 1, the four categories of cases showed similar survival intervals (within 2 h). The cases from the SCD-positive autopsy group had significant increases in the heart weight as compared with both control cases (*p* < 0.001). The heart weight of the SCD-negative autopsy group was, however, significantly lower than that of the SCD-positive autopsy group (*p* < 0.05), though it was still higher than both control cases (Table 1). Furthermore, SCD-negative autopsy cases were not identified of any gross cardiovascular change as similar with control cases, while 56.2% (*n* = 9) of SCD-positive autopsy cases showed coronary stenosis (50–75% stenosis), calcification and myocardial fibrosis, and the remaining 43.8% (*n* = 7) showed coronary stenosis (> 75%), severe myocardial fibrosis and disorder (Table 2 and Fig. 1). The myocardium from the SCD-positive autopsy group was disarrayed with a halo of fibrotic tissues within the degenerated myocardium (Fig. 1). In consistent with the short survival time, 88.9% of SCD-negative autopsy cases showed histological evidence of acute myocardial ischemia (within 2 h) such as interstitial edema, wavy fibers, and hypereosinophilia of cardiomyocytes (Table 2). No evident fibrotic tissue was observed for the SCD-negative autopsy group (Fig. 1).

### Common molecular markers failed to discriminate the SCD cases categorized as SCD-negative autopsy from control cases

**Fig. 2:**
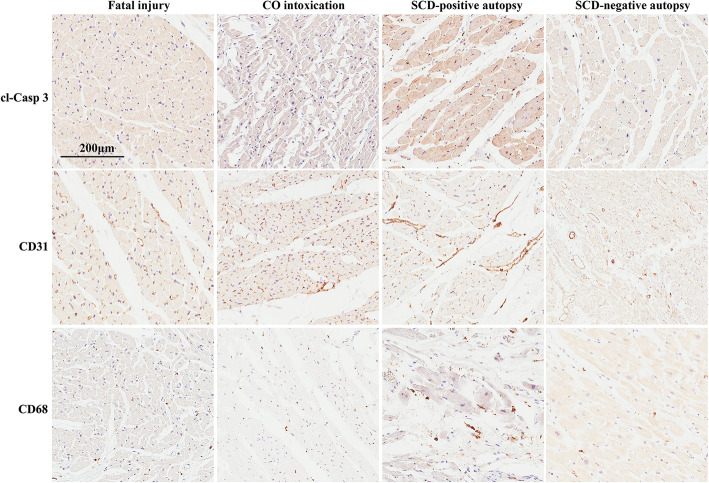
Representative photographs of cleaved-Caspase3 (cl-Casp3), CD31, and CD68 expression in heart tissues stained by immunohistochemistry. SCD-positive autopsy group showed increases in the expression of cl-Casp3 (cytoplasm positivity in apoptotic cells), CD31 (lumen positivity), and CD68 (a marker of macrophage), whereas fatal injury and CO intoxication cases were absent from strong positivity of the above markers. SCD-negative autopsy cases showed patchy positivity of cl-Casp3 and dim CD31 signal, and were absent of CD68 positivity. Scale bar = 200 μm

To further examine molecular alterations in the heart samples of SCD, we assayed the common molecular biomarkers of myocardial injury, including cleaved-Caspase3 (cl-Casp3), CD31, and CD68. Cl-Casp3 is a marker of early myocardial injury and cell apoptosis. CD68 is a marker of macrophage infiltration which often occur one or two days after injury. CD31 is involved in angiogenesis, leukocyte transmigration and integrin activation, and the changes of CD31-positive cells are commonly observed during cardiac remodeling [29]. As expected, the heart samples from the SCD-positive autopsy group showed notable positivity of cl-Casp3 and CD68. Neovascularization was also evident in the SCD-positive autopsy group as by IHC staining of CD31. The SCD-negative autopsy group showed patchy positivity of cl-Casp3 and dim CD31 signal, and was absent of CD68 positivity. These changes were milder as compared with the SCD-positive autopsy group, though they were to some extent pathological as compared with the fatal injury and CO intoxication cases that were consistently absent of cl-Casp3, CD31, and CD68 positivity (Fig. 2). These data suggested that SCD-negative autopsy was hard to be discriminated using the histological and IHC assays.

### miR-3113-5p, miR-223-3p, miR-499a-5p, and miR-133a-3p significantly upregulated in the SCD

**Fig. 3:**
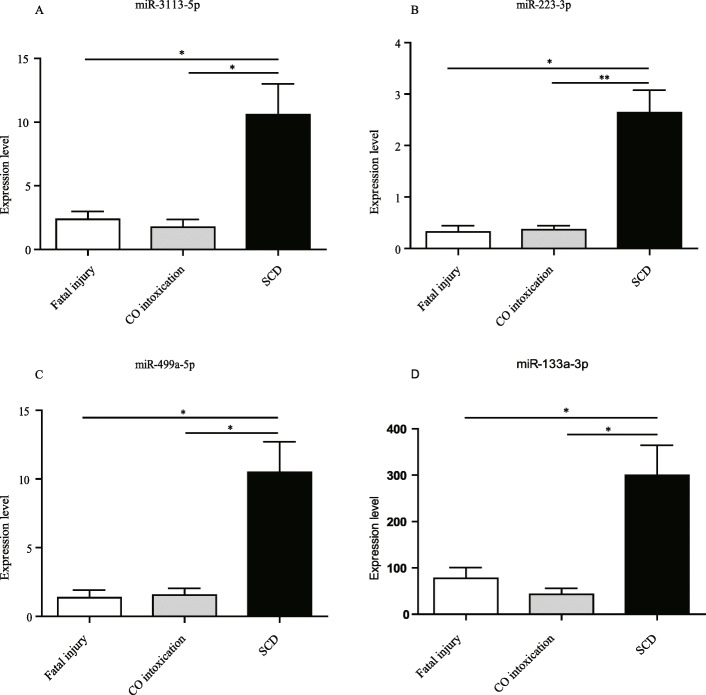
The relative expression level of the four miRNAs among fatal injury (*n* = 14), CO intoxication (*n* = 14) and SCD groups (*n* = 34). The expression of miR-3113-5p (A), miR-223-3p (B), miR-499a-5p (C) and miR-133a-3p (D) were detected by RT-qPCR. Results were normalized with U6 gene, *p < 0.05, **p < 0.01

In view that common histological and IHC techniques could not aid in diagnosing SCD-negative autopsy, we speculated that small-molecules might be helpful. We thus assessed whether four cardio-miRNAs (miR-3113-5p, miR-223-3p, miR-499a-5p, and miR-133a-3p) had any diagnostic values. The selection of these miRNAs was based on published literature [28, 30, 31] and our previous work [26] . Our aims were two-fold: one to assess whether these miRNAs could diagnose SCD, and the other one to assess whether specific miRNAs had higher efficacy as to discriminate the causes of SCD. To this end, we initially pooled the SCD-positive autopsy and SCD-negative autopsy groups as SCD cases. RT-qPCR was performed with U6 as a stable reference gene. Our results revealed that miR-3113-5p was significantly elevated by approximate five-fold in the SCD groups (Fig. 3A). miR-223-3p and miR-499a-5p were also increased by approximate five-fold (Fig. 3B and C). miR-133a-3p showed significant increases for approximate three-fold when compared to both controls (Fig. 3D).

### miR-3113-5p, miR-223-3p, miR-499a-5p, and miR-133a-3p could aid in diagnosing SCD

**Fig. 4:**
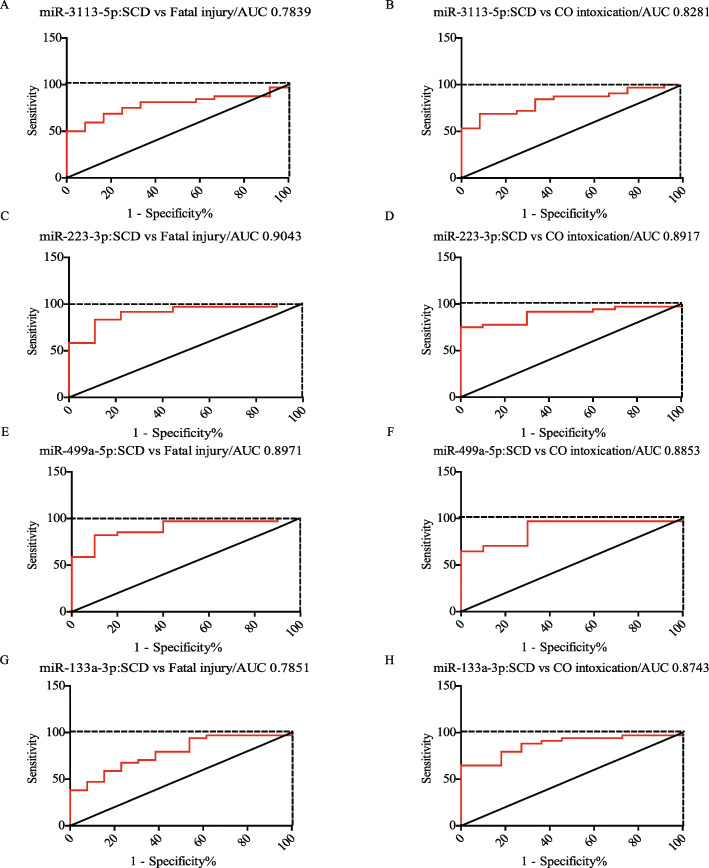
Diagnostic performance of the four miRNAs were analyzed by ROC curve. (A, B) ROC curve of miR-3113-5p. (C, D) ROC curve of miR-223-3p. (E, F) ROC curve of miR-499a-5p. (G, H) ROC curve of miR-133a-3p

We next assessed the diagnostic values of these miRNAs for distinguishing SCD from non-cardiac deaths. Receiver operating characteristic (ROC) curve analysis showed that the area under the curve (AUC) of miR-3113-5p were 0.7893 (SCD vs Fatal injury: 95% confidence interval (CI) = 0.6501 to 0.9176, sensitivity = 68.75% and specificity = 83.33%) and 0.8281 (SCD vs CO intoxication: 95% CI = 0.7071 to 0.9492, sensitivity = 68.75% and specificity = 91.67%) (Fig. 4A and B). The AUCs of miR-223-3p were 0.9043 (SCD vs Fatal injury: 95% CI = 0.8031 to 1.006, sensitivity = 83.33% and specificity = 88.89%) and 0.8917 (SCD vs CO intoxication: 95% CI = 0.7984 to 0.9849, sensitivity =75% and specificity = 100%) (Fig. 4C and D). The AUCs of miR-499a-5p were 0.8971 (SCD vs Fatal injury: 95% CI = 0.7972 to 0.9969, sensitivity = 82.35% and specificity = 90%) and 0.8853 (SCD vs CO intoxication: 95% CI = 0.7771 to 0.9935, sensitivity =97.06% and specificity = 70%) (Fig. 4E and F). The AUCs of miR-133a-3p were 0.7851 (SCD vs Fatal injury: 95% CI = 0.6466 to 0.9235, sensitivity = 67.65% and specificity =76.92%) and 0.8743 (SCD vs CO intoxication: 95% CI = 0.7693 to 0.9793, sensitivity =64.71% and specificity = 100%), respectively (Fig. 4G and H). Detailed ROC analysis results were listed in Table 4. These results demonstrated that miR-3113-5p, miR-223-3p, miR-499a-5p, and miR-133a-3p could be used as sensitive biomarkers for diagnosis of SCD.

**Table 4:** Receive operating characteristic (ROC) analysis of miRNAs

	AUC	Std.Error	***P*** value	LB(95%)	UB(95%)	Sensitivity	Specificity	Youden index
miR-3113-5p SCD vs Fatal injury	0.7839	0.06823	0.004091	0.6501	0.9176	68.75%	83.33%	0.52
miR-3113-5p SCD vs CO intoxication	0.8281	0.06175	0.0009054	0.7071	0.9492	68.75%	91.67%	0.60
miR-223-3p SCD vs Fatal injury	0.9043	0.05161	0.0002037	0.8031	1.006	83.33%	88.89%	0.72
miR-223-3p SCD vs CO intoxication	0.8917	0.04755	0.0001753	0.7984	0.9849	75%	100%	0.75
miR-499a-5p SCD vs Fatal injury	0.8971	0.05094	0.0001582	0.7972	0.9969	82.35%	90%	0.72
miR-499a-5p SCD vs CO intoxication	0.8853	0.05518	0.0002463	0.7771	0.9935	97.06%	70%	0.67
miR-133a-3p SCD vs Fatal injury	0.7851	0.07063	0.002743	0.6466	0.9235	67.65%	76.92%	0.45
miR-133a-3p SCD vs CO intoxication	0.8743	0.05356	0.0002201	0.7693	0.9793	64.71%	100%	0.65

*AUC* Area under the curve, *LB* Lower bound, *UB* Upper bound

### miR-3113-5p, miR-223-3p, miR-499a-5p, and miR-133a-3p were significantly up-regulated in the SCD-negative autopsy group compared with the SCD-positive autopsy group

**Fig. 5:**
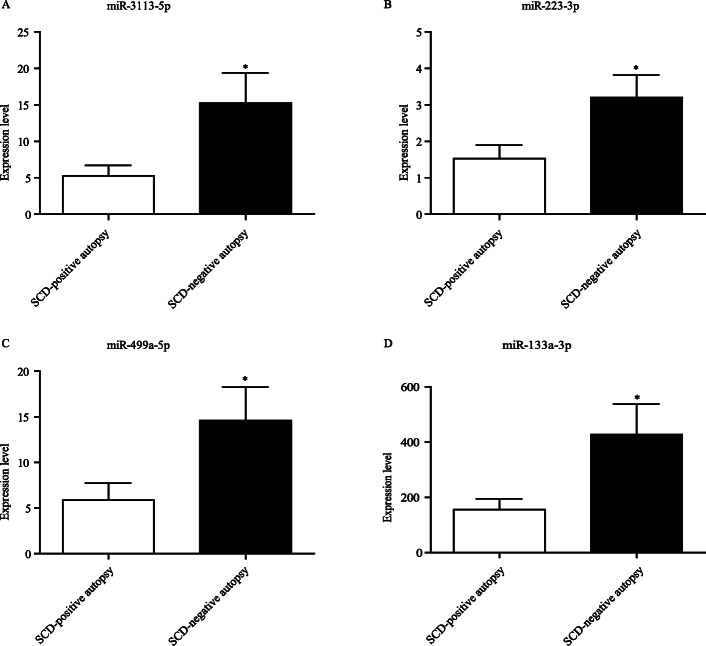
The relative expression level of the four miRNAs between SCD-positive autopsy (*n* = 18) and SCD-negative autopsy groups (*n* = 16). miR-3113-5p (**A**), miR-223-3p (**B**), miR-499a-5p (**C**), and miR-133a-3p (**D**) were upregulated in SCD-negative autopsy group as compared to the SCD-positive autopsy. Results were normalized to U6 gene, *p < 0.05 versus SCD-positive autopsy

To precisely evaluate the power of the four miRNAs in predicting SCD, SCD was divided into SCD-positive autopsy (with gross coronary artery stenosis) and SCD-negative autopsy (without gross heart changes). Meanwhile, the expression patterns of the four miRNAs were detected in SCD-positive autopsy and SCD-negative autopsy groups by RT-qPCR. Interestingly, miR-3113-5p (Fig. 5A), miR-223-3p (Fig. 5B), miR-499a-5p (Fig. 5C), and miR-133a-3p (Fig. 5D) were all significantly higher in the SCD-negative autopsy group than those in the SCD-positive autopsy group with the fold-change ranging from two to three (*p* < 0.05).

### Diagnostic efficiency of the miRNAs in discriminating the detailed causes of SCD

**Fig. 6:**
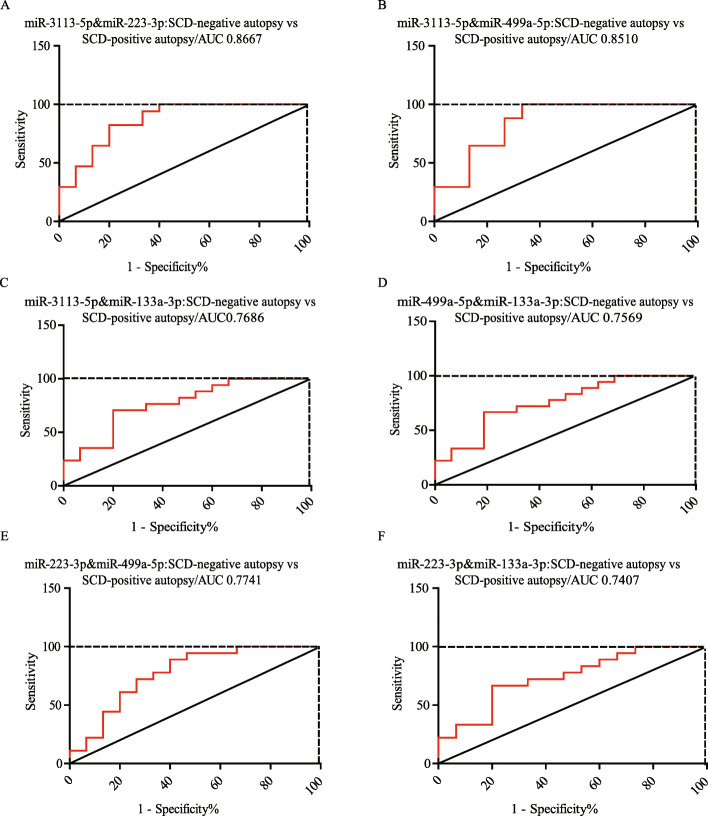
Diagnostic performance of miRNAs was analyzed by ROC curve. The ROC analysis for detection of SCD-positive autopsy and SCD-negative autopsy using a combination of miR-3113-5p and miR-223-3p (**A**), a combination of miR-3113-5p and miR-499a-5p (**B**), a combination of miR-3113-5p and miR-133a-3p (**C**), a combination of miR-499a-5p and miR-133a-3p (**D**), a combination of miR-223-3p and miR-499a-5p (**E**), a combination of miR-223-3p and miR-133a-3p (**F**) were shown

Next, ROC curve analyses were used to assess the diagnostic ability of the four miRNAs for discriminating SCD-negative autopsy from SCD-positive autopsy. It revealed that neither of these miRNAs alone efficiently discriminated SCD-negative autopsy (AUC was 0.7373 for miR-3113-5p, AUC was 0.7200 for miR-223-3p, AUC was 0.7569 for miR-133a-3p, and AUC was 0.7951 for miR-499a-5p), indicating that SCD-negative autopsy and SCD-positive autopsy both shared common pathologic basis, albeit SCD-negative autopsy occurring in a sudden and unidentified way. We then assessed the ROC by combing these miRNAs. Our results showed that the AUC of a combination of miR-3113-5p and miR-223-3p was 0.8667 (95% CI = 0.7388 to 0.9945, sensitivity = 82.35% and specificity =80%, Fig. 6A). The AUC of a combination of miR-3113-5p and miR-499a-5p was 0.8510 (95% CI = 0.7105 to 0.9915, sensitivity = 100% and specificity =66.67%, Fig. 6B). The AUC of a combination of miR-3113-5p and miR-133a-3p was 0.7686 (95% CI = 0.6032 to 0.9341, sensitivity =70.59% and specificity =80%, Fig. 6C). The AUC of a combination of miR-499a-5p and miR-133a-3p was 0.7569 (95% CI = 0.5938 to 0.9201, sensitivity = 66.67% and specificity =81.25%, Fig. 6D). The AUC of a combination of miR-223-3p and miR-499a-5p was 0.7741 (95% CI = 0.6062 to 0.9419, sensitivity = 88.89% and specificity =60%, Fig. 6E). The AUC of a combination of miR-223-3p and miR-133a-3p was 0.7407 (95% CI = 0.5700 to 0.9115, sensitivity = 66.67% and specificity =80%, Fig. 6F). The details were shown in Table 5. These results demonstrated that miR-3113-5p and miR-223-3p together yielded the highest diagnostic performance in discriminating SCD-negative autopsy from SCD-positive autopsy (maximum of AUC). miR-3113-5p and miR-499a-5p together also showed high AUC values and sensitivity (maximum of Youden’s index).

**Table 5:** Receive operating characteristic (ROC) analysis of miRNAs

	AUC	Std.Error	P value	LB(95%)	UB(95%)	Sensitivity	Specificity	Youden index
miR-3113-5p & miR-223-3p	0.8667	0.06520	0.0004180	0.7388	0.9945	82.35%	80%	0.62
miR-3113-5p & miR-499a-5p	0.8510	0.07165	0.0007310	0.7105	0.9915	100%	66.67%	0.67
miR-3113-5p & miR-133a-3p	0.7686	0.08438	0.009717	0.6032	0.9341	70.59%	80%	0.51
miR-499a-5p & miR-133a-3p	0.7569	0.08320	0.01070	0.5938	0.9201	66.67%	81.25%	0.48
miR-223-3p & miR-499a-5p	0.7741	0.08562	0.007487	0.6062	0.9419	88.89%	60%	0.49
miR-223-3p & miR-133a-3p	0.7407	0.08709	0.01881	0.5700	0.9115	66.67%	80%	0.47

*AU*C Area under the curve, *LB* Lower bound, *UB* Upper bound

## Discussion

Cardiovascular disease is still the main cause of morbidity and mortality worldwide. miRNAs play important roles in regulating cardiac development, remodeling and regeneration, endothelial function, vasculogenesis and neoangiogenesis through a variety of pathways [32]. Aberrant miRNA expression profiles are associated with various cardiovascular conditions such as hypertrophy, fibrosis, heart failure, and arrhythmias [33]. Studies have shown that miRNAs are circulating freely in mammalian blood with marked biostability and can be detected with high sensitivity and specificity in human plasma and serum [34]. So, the diagnostic potential of miRNA detection in human plasma for cardiovascular disorders was to be explored. Coronary artery disease is the main cause of SCD. It was reported that the circulating miR-1 [35] and miR-208a [36] significantly increased in AMI patients compared to non-AMI controls, indicating miR-1 and miR-208a in serum may serve as biomarkers for AMI. miR-1 is also upregulated in the heart from patients with coronary artery disease and in rat ischemic hearts, which correlates to an increase in arrhythmogenesis [37]. miR-135a increased and miR-147 decreased significantly in plasma of atherosclerotic cardiovascular diseases patients, and miR-135a/miR-147 ratio could be used for atherosclerotic cardiovascular diseases diagnosis [38]. Circulating miR-106a may function as potential biomarker in patients with coronary artery disease [39]. Although there has been many researches focusing on circulating/plasma biomarkers to enhance SCD risk prediction and diagnostic performance, few cardio-miRNAs have previously been identified, and none of these biomarkers have progressed to clinical use for SCD. Thus, there remains a critical need to identify biomarkers of SCD.

Several studies have reported that miR-223 is associated with myocardial diseases. miR-223-3p inhibits ischemia-reperfusion induced cardiac necroptosis at multiple layers which may constitute a new therapeutic agent for the treatment of ischemic heart disease [30]. miR-223 was also shown to be downregulated in ET-1 induced hypertrophic myocardium by targeting TNNI3K [40]. miR-223 was upregulated in a rat model of AMI and its overexpression promoted cardiac arrhythmias [23]; thus, miR-223 may be a new target in the treatment of ischemic arrhythmias. Our previous research showed that cardiac miR-3113-5p might be a useful target for therapeutic purposes and circulating miR-3113-5p might serve as a stable marker for early diagnosis of cardiac ischemia-reperfusion injury [26]. miR-499-5p is highly conserved and preferentially expressed in the myocardium [41]; circulating levels of miR-499-5p were significantly higher at admission in AMI patients that died within the following year as compared to those who survived the cardiovascular event [42]. Overexpression of miR-499-5p in cardiomyocytes was able to protect the heart against myocardial infarction-associated tissue damage in vivo [43]. A study reported that miR-133a-3p inhibits cardiomyocyte apoptosis by targeting caspase-9 [44], and miR-133a-3p has been implicated in the control of myocardial fibrosis [45]. The above reports suggested these microRNAs had clinical importance in the diagnosis of SCD.

Many studies have recommended histological and IHC markers to be applied in the diagnosis of myocardial damage [46–48]. Pertaining to the early myocardial damage, IHC staining has particular advantages due to their useful application in FFPE tissues and in assessing ischemic areas during acute myocardial ischemia [46]. Unfortunately, a single immunohistochemical marker often cannot meet the requirements of diagnosis when no macroscopic or microscopic evidence of necrosis is available, and the IHC staining may be inevitably affected by sampling bias and interpretation controversy [46–48]. In this study, immunohistochemical markers (cell apoptotic marker cl-Casp3, angiogenesis marker CD31, and macrophage infiltration marker CD68) were found to not aid in diagnosing SCD-negative autopsy. Small-molecules, namely the four cardio-miRNAs (miR-3113-5p, miR-223-3p, miR-499a-5p, and miR-133a-3p) were, however, found to be useful to diagnose SCD in human real cases. Our study demonstrated that miR-3113-5p, miR-223-3p, miR-499a-5p and miR-133a-3p may serve as candidate diagnostic biomarkers for SCD, allowing to distinguish subjects with SCD from others. The expression levels of miR-3113-5p, miR-223-3p, miR-499a-5p and miR-133a-3p were significantly up-regulated in the heart tissues from SCD as compared to control subjects. The ROC analysis revealed that the four miRNAs may serve as independent diagnostic markers of SCD. Among the miRNAs, miR-223-3p and miR-499a-5p showed better accuracy with the highest AUC values. In particular, we further found that several miRNA panels consisting two of the four miRNAs could achieve better discriminative capacity when discriminating the causes of SCD. These results showed that miR-3113-5p, miR-223-3p, miR-499a-5p, and miR-133a-3p may be considered as potential candidate biomarkers for diagnosis of SCD.

Previous studies have focused on circulating miRNAs that may be novel biomarkers for SCD-negative autopsy or SCD. For example, an elevated level of circulating miR-133a may serve as a diagnostic marker of AMI [49]. Circulating miR-499 and miR-223 can effectively differentiate AMI from non-AMI patients [50]. Unfortunately, these studies mainly focused on plasma or serum samples. The role of these miRNAs at their origin (cardiac-derived miRNAs, cardio-miRNAs) remained largely unknown. To this end, we used human heart samples to evaluate these cardio-miRNAs in SCD cases. Archival FFPE heart tissue samples are readily available resources for miRNA biomarker identification [51]. It has been demonstrated that miR-1, miR-499, miR-133, and miR-208 are sensitive markers to diagnose SCD-negative autopsy and SCD using post-mortem FFPE heart tissues [28], an observation that was also confirmed by the current study. In addition, our study represents the first one, to the best knowledge of us, to report the expression profiling of miR-3113-5p and miR-223-3p in FFPE heart tissues, and extended their biological importance as a diagnostic biomarker for SCD.

For a long time, the study of miRNAs could only be performed in model animals which was defected from clinical and forensic reality. The present study used FFPE tissues as a novel research material to maximally promote the findings conforming with human bodies. FFPE samples are valuable research materials, stored in pathology archives worldwide. Storing FFPE specimens is more economical than storing frozen samples, and the histological structure can be preserved almost permanently [51]. However, formalin fixation and paraffin embedding inevitably lead to nucleic acids degradation in these tissues. DNA and RNA are fragmented and chemically modified [52], which sometimes causes inconsistency between the results obtained using matched fresh and FFPE samples [53]. Fortunately, miRNAs are robust and stable in FFPE heart tissues, which can be utilized as deep sequencing and quantitative qPCR analyses [54]. A study has suggested that miRNAs remain highly stable in postmortem FFPE heart tissues, and validated that miR-1, miR-208b, and miR-499a in FFPE heart tissues are diagnostic biomarkers for SCD-negative autopsy patients [28]. Therefore, we believe that the use of FFPE as a clinic relevant material opens novel insights into disease pathogenesis, particularly with regard to the identification of miRNAs.

## Conclusions

Our study showed the potential of miR-3113-5p, miR-223-3p, miR-499a-5p, and miR-133a-3p as candidate biomarkers for diagnosis of SCD. We propose that individual of miR-3113-5p, miR-223-3p, miR-133a-3p, and miR-499a-5p can serve as sensitive biomarkers to diagnose SCD, and a pool of these miRNAs could be further used to discriminate the causes of SCD, particularly the cases without positive autopsy findings.

## Data Availability

All data generated or analyzed during this study are included in this published article.

## Abbreviations

SCD: Sudden cardiac deaths
AMI: Acute myocardial infarction
SCD-positive autopsy: Sudden cardiac death with positive autopsy
SCD-negative autopsy: Sudden cardiac death with negative autopsy
CO: Carbon monoxide
ROC: Receiver operating characteristic
AUC: Area under the curve
CI: Confidence interval
miRNA: MicroRNA
OR: Odds ratio
H&E: Hematoxylin & Eosin
PSR: PicroSirius Red
FFPE: Formalin-fixed paraffin-embedded
IHC: Immunohistochemistry
IHC: Horseradish peroxide
LB: Lower bound
UB: Upper bound
